# Predicting outcomes in primary spontaneous pneumothorax using air leak measurements

**DOI:** 10.1136/thoraxjnl-2018-212116

**Published:** 2018-10-24

**Authors:** Rob J Hallifax, Magda Laskawiec-Szkonter, Najib M Rahman

**Affiliations:** 1 Oxford Centre for Respiratory Medicine, Oxford University Hospitals NHS Trust, Oxford, UK; 2 Oxford Respiratory Trials Unit, University of Oxford, Oxford, UK; 3 NIHR Oxford Biomedical Research Centre, University of Oxford, Oxford, UK

**Keywords:** pleural disease, respiratory measurement

## Abstract

The initial treatment regime for primary spontaneous pneumothorax (PSP) is generic and non-personalised, often involving a long hospital stay waiting for air leak to cease. This prospective study of 81 patients with PSP, who required drain insertion, captured daily digital air leak measurements and assessed failure of medical management against prespecified criteria. Patients with higher air leak at day 1 or 2 had significantly longer hospital stay. If air leak was ≥100 mL/min on day 1, the adjusted OR of treatment failure was 5.2 (95% CI 1.2 to 22.6, p=0.03), demonstrating that early digital air leak measurements could potentially predict future medical treatment failure.

**Trial registration number:**

ISRCTN79151659.

## Introduction

Spontaneous pneumothorax is a common pathology with an incidence of 17–24 and 1–6 per 100 000 population per annum for men and women, respectively.^1 2^ Primary spontaneous pneumothorax (PSP) conventionally refers to patients with no underlying lung disease.

The optimal initial treatment regime for PSP is not yet defined. Options include conservative management, needle aspiration (NA) or insertion of a small-bore chest drain. International guidelines and expert consensus statements vary.^3–5^ The British Thoracic Society (BTS) guidelines suggest treatment is required in patients with a large pneumothorax and/or symptoms.^3^ NA of up to 2.5 L of air is recommended as initial treatment. If aspiration is unsuccessful, then chest tube insertion is required.

Ideally, removal of the air from the pleural cavity will allow the lung to re-expand. This relies on the air leak, which occurs via a hole in the visceral pleura (ie, an alveolar–pleural fistula), having ceased. Standard management currently involves daily monitoring for ongoing air leak, using bubbling of the chest tube as a marker. Although ambulatory strategies have been described, a meta-analysis showed these data to be highly bias (a large number of case series) and outlined the need for a randomised controlled trial.^6^ This is the rationale behind the currently recruiting Randomised Ambulatory Management of Primary Spontaneous Pneumothorax (RAMPP) trial (see below), but currently, the practice remains to treat patients as inpatients in hospital. This management strategy is unsatisfactory for both patients and clinicians; bubbles via the chest drain are not precise (they are not consistently present) and are a binary measure which do not quantify the degree of air leak.

Digital suction devices, such as the Thopaz+ (Medela, Switzerland), provide a quantitative measure of airflow. These devices are widely used post-thoracic surgery in the UK and have been shown to reduce chest drain duration and hospital stay.^7–9^

We hypothesise that early quantification of air leak predicts those patients who will have prolonged air leak and hence failed medical management (requiring surgical referral).

## Methods

Data was a preplanned interim subgroup analysis from the RAMPP trial comparing standard care with an entirely ambulatory management strategy using an integrated Pleural Vent device (Rocket Medical, UK) (see ISRCTN 79151659). Patients were randomised to either to standard chest tube (12 French gauge, Fg) or Pleural Vent (8 Fg). The outcome of interest was the failure of medical management was based on prespecified criteria (based on BTS guidelines^3^) of: persistent bubbling via standard chest drain and/or unexpanded lung at day 4 postdrain insertion. The digital air leak measurement did not form part of decision-making.

Digital airflow measurements were taken by attaching the Thopaz+ device to either chest drain or Pleural Vent for 10 min daily until day 4, or drain removal. The patency of the chest tube was always checked prior to measurement. The Thopaz+ device was set to −0.4 kPa as close as possible to physiological intrapleural pressure (which at functional residual capacity is −0.3 to −0.5 kPa in health) thereby passively measuring air flow, rather than actively providing suction.

This preplanned analysis was intended to be exploratory. Therefore, three air leak levels were modelled based on previous experience (at 50, 100 and 150 mL/min). Other *a priori* risk factors assessed using logistic regression were: sex, size of initial pneumothorax on chest radiograph (CXR), smoking status and body mass index (BMI). Comparison of means were made by t-test, medians by Mann-Whitney U-test and proportions in each group (ORs) by Χ^2^ test.

## Results

This analysis included 81 patients with PSP: 60 (74.1%) were male, mean age 30.0 years (SD 7.9), 74.1% were current or ex-smokers and 25.9% never smokers. Twenty patients (24.7%) met criteria for failure of medical management.

### Air leak

The median air leak was greater in patients failing medical management (vs spontaneous resolvers) on day 1: 360 versus 10 mL/min (p=0.02) and day 2: 190 vs 10 mL/min (p=0.03).

**Figure 1:**
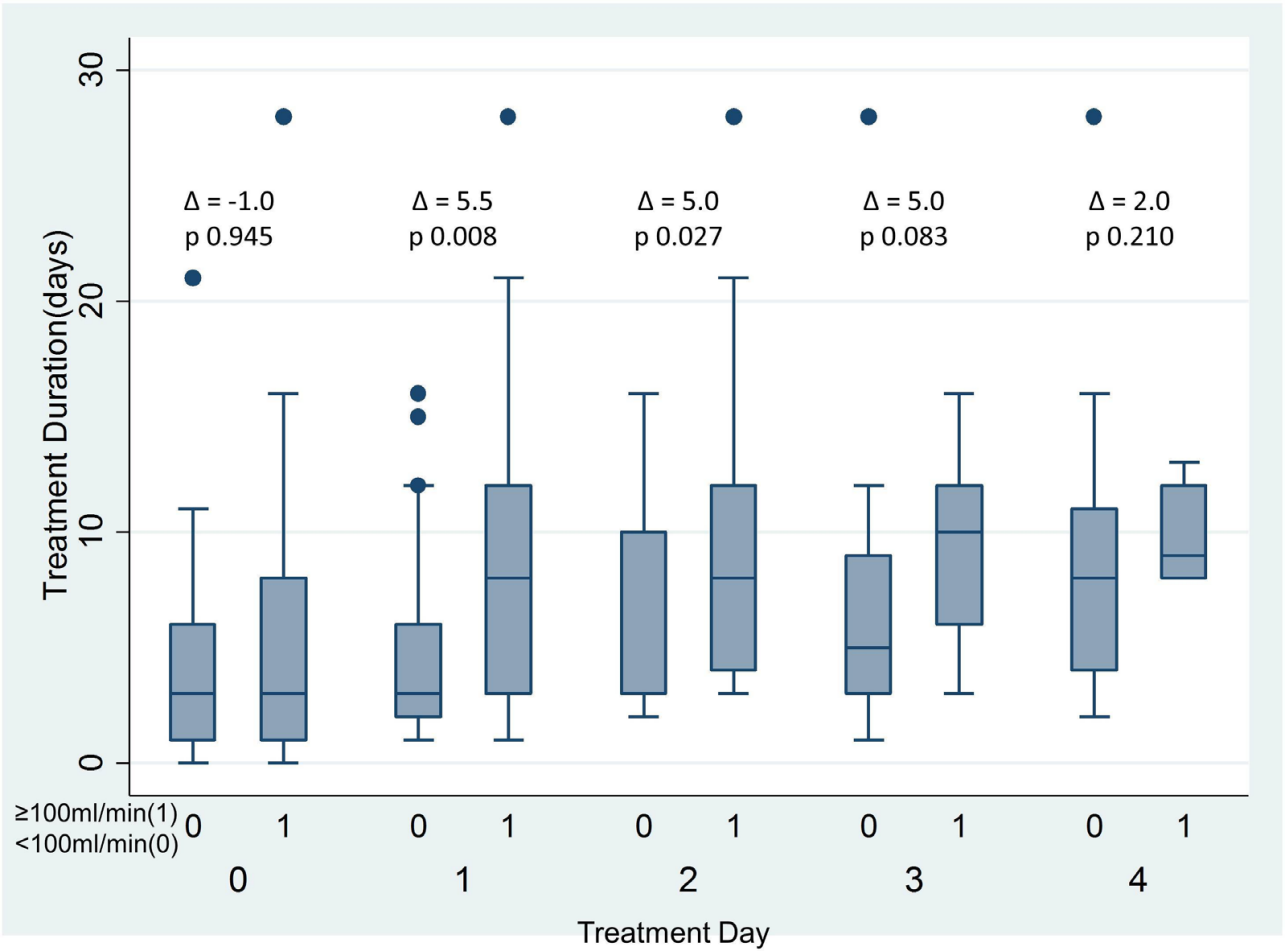
Median treatment duration (days) by air leak threshold (100 mL/min) by treatment day. Δ=difference in medians. P values calculated by Mann-Whitney U-test). Bars represent medians and IQR. Dots represent data outside the IQR.

Patients with a greater air leak at day 1 or 2 had significantly longer hospital stay (see figure 1). A value of an air leak ≥100 mL/min was associated with failure of medical management, as per prespecified criteria. If the air leak was ≥100 mL/min on day 1, the unadjusted OR of failure of medical management was 5.2 (95% CI 1.3 to 20.0, p=0.01) (see table 1). The negative predictive value of air leak <100 mL/min at day 1 was 80.6%; that is, only one in five patients with an air leak at this level failed to resolve spontaneously (ie, without recourse to surgical repair). There were no differences in patient characteristics by air leak category (see online Supplementary appendix).

10.1136/thoraxjnl-2018-212116.supp1 Supplementary data

**Table 1:** Odd Ratios (OR) of failure of medical management by patient characteristics: univariate and multivariable logistic regression

Factor	Univariate (unadjusted)		Multivariable (adjusted)	
	OR (95% CI)	P values	OR (95% CI)	P values
Sex (F:M)	1.8 (0.6 to 5.5)	0.29	2.6 (0.4 to 15.3)	0.29
Size (large:small)	1.4 (0.4 to 4.3)	0.60	0.9 (0.1 to 5.5)	0.90
Smoker (ever:never)	0.4 (0.1 to 1.2)	0.10	0.4 (0.1 to 2.2)	0.28
Body mass index (kg/m^2^) (≤18.5:>18.5)	3.1 (0.7 to 13.7)	0.12	2.0 (0.3 to 14.9)	0.50
D1 Air leak≥100 mL/min*	5.2 (1.3 to 20.0)	0.01	5.2 (1.2 to 22.6)	0.03

*Measurement on day 1.

### Other risk factors

Patients with large pneumothoraces (≥4 cm at hilum) were not statistically more likely to be fail medical management (OR 1.4 (95% CI 0.4 to 4.3), p=0.60) or to have longer treatment duration (mean 5.4 days (SD 5.5) vs 3.6 days (SD 4.7), p=0.20). Multivariable regression demonstrated that the adjusted OR of medical management failure were not significantly higher for underweight patients (BMI <18.5 kg/m^2^), female sex and for non-smokers; the only independent risk factor was air leak ≥100 mL/min on day 1 (see table 1).

## Discussion

This is the first study to use sequential digitally measured airflow in PSP as a predictor of medical treatment failure, using a standardised treatment protocol and prespecified criteria to define treatment failure. Historically, Seaton *et al* devised a direct test of air leak at NA by asking patients to inhale chlorofluororcarbon (CFC) gas, while their pneumothorax was aspirated. If the CXR had improved and no CFC gas was detected, 96% required no further treatment; compared with 51% if CFC gas was detected.^10^ This technique has not been adopted clinically, because of impracticality and lack of availability of CFC inhalers. Therefore, guidelines advise a generic management paradigm.

Our data demonstrates that digital air leak measurements early in the treatment course potentially predict future treatment failure. A limitation of this study is that air leak measurements were taken via either standard chest drain or Pleural Vent. However, the patients were randomised, so any systematic differences in drain length and gauge (30 cm 12 Fg drain vs 11 cm 8 Fg Pleural Vent) should be eliminated. Further prospective data collection is ongoing as part of the RAMPP study; if this hypothesis is validated, early prediction of treatment failure could lead to a streamlined treatment pathway. Patients at high risk of failure could be triaged to early thoracic surgery rather than waiting with daily reviews until day 4, avoiding the associated costs of inpatient stay in addition to patient uncertainty. This new treatment paradigm will need to be assessed prospectively in a controlled trial, but has the potential to change the management strategy for acute pneumothorax for the first time in decades.
