# Supplementary material for: Predicting outcomes in primary spontaneous pneumothorax using air leak measurements
Source: Thorax. 2018 Oct 24;74(4):410–2. doi: 10.1136/thoraxjnl-2018-212116 (PMC6475109; doi:10.1136/thoraxjnl-2018-212116)
Supplement: Supplementary data [file thoraxjnl-2018-212116supp001.docx]

Appendix Table: Demographics of patients by air leak threshold (< and >=100ml/min)

|  |  | **Air leak threshold** | |
| --- | --- | --- | --- |
| **Demographic** |  | <100ml/min | ≥100ml/min |
|  |  |  |  |
| Male (%) |  | 62.5% | 77.8% |
| Age (mean, SD) |  | 29.4 (6.1) | 30.5 (7) |
| % large pneumothorax |  | 83.3% | 66.7% |
| % Smoker (ex or current) | | 62.5% | 77.8% |
| % BMI <=18.5 |  | 8.3% | 22.2% |
